# Facile Fabric Detoxification Treatment Method Using Microwave and Polyethyleneimine Against Nerve Gas Agents

**DOI:** 10.3390/polym12122861

**Published:** 2020-11-30

**Authors:** Woong Kwon, Changkyu Kim, Jiyun Kim, Jongwon Kim, Euigyung Jeong

**Affiliations:** 1 Department of Textile System Engineering, Kyungpook National University, Daegu 41566, Korea; kwoong7242@naver.com (W.K.); se02126000@gmail.com (C.K.); k1m.j2.y00n@gmail.com (J.K.); 2 Department of Fiber System Engineering, Yeungnam University, Gyeongsan 38541, Korea

**Keywords:** polyethyleneimine, detoxification fabrics, nerve agent, diisopropylfluorophosphate

## Abstract

Generally, detoxification fabrics are defined as fabrics that remove or inhibit the production of toxic compounds, especially chemical warfare agents such as nerve gas agents. They are usually prepared using a complicated and time-consuming method. This study suggests a facile treatment method for preparing detoxification fabrics against nerve gas agents using polyethyleneimine and microwave curing. The detoxification properties of polyethyleneimine and microwave-treated polypropylene nonwoven fabric were evaluated using diisopropylfluoro-phosphate, which is a nerve agent simulant. The treated polypropylene fabric decontaminated 53.6% of diisopropylfluorophosphate (DFP) in 2 h at 32 °C, and the half-life of DFP on the surface of the treated fabric was 122 min. The result indicates that the treated fabric can act as a basic organocatalyst for the DFP hydrolysis and has a shorter half-life owing to the large number of amine groups. Therefore, the facile treatment method has the potential for use in the preparation of detoxification fabrics.

## 1. Introduction

Chemical warfare agents (CWAs) are extremely toxic compounds and can be dangerous when inhaled or when they come into contact with the human body. Nerve agents are considered to be the most toxic compounds of CWAs. They bind to the active site of acetylcholinesterase, which results in the inhibition of acetylcholine breakdown, thus leading to the buildup of acetylcholine and damaging the human body [1,2,3,4]. Nerve agents are organic compounds with phosphorus–sulfur (P–S) or phosphorus–fluorine (P–F) bonds, which are slowly hydrolyzed to P–OH bonds, resulting in the loss of toxicity [5,6]. 

Detoxification fabrics that are capable of detoxifying nerve agents have been investigated to protect the human body against nerve agents. They are prepared using catalyst materials, which can facilitate the hydrolysis of nerve agents. Catalyst materials, including metal–organic frameworks (MOF), transition metal catalysts, and basic organic compounds [7,8,9,10,11,12,13,14], are applied to fabrics, and the detoxification properties are evaluated using diisopropylfluorophosphate (DFP), a simulant nerve agent, instead of the actual nerve agents. DFP is hydrolyzed to diisopropyl hydrogen phosphate (DHP), and the detoxification properties are determined by converting the amount of DFP [7,8,9,10,11,12,13,14]. 

Ying et al. reported the synthesis of guanidine-functionalized poly[2-(3-butenyl)-2-oxazoline] (G-PBuOxz) with the thiol-ene click reaction and guanidinylation to prepare detoxification fabrics. Detoxification fabrics were prepared via co-electrospinning using a mixture of nylon and G-PbuOxz. However, the co-electrospun web revealed that 100% of DFP was hydrolyzed to DHP in 2 h at 32 °C [7]. Choi et al. reported the synthesis of guanidine-functionalized thermoplastic polyurethane with the azide-alkyne click reaction and guanidinylation to prepare detoxification fabrics. The guanidine-functionalized thermoplastic polyurethane exhibited only 51% DFP hydrolysis in 2 h at 32 °C [8]. Lee et al. reported that basic groups such as ethylenediamine, diethylenetriamine, and triethylene-tetramine had been introduced into polyacrylonitrile (PAN) fabrics via chemical vapor deposition (CVD) to prepare detoxification fabrics. Moreover, PAN fabrics, which have basic groups, could hydrolyze 10–37% of DFP in 2 h at 32 °C [9]. Kim et al. reported that zirconium hydroxide was coated on nylon using the sol–gel method to prepare detoxification fabrics, which exhibited only 40% of DFP hydrolysis to DHP [10]. In another report, Kwon et al. prepared detoxification fabrics by treating a cotton fabric with guanidinylated chitosan (Gu-chitosan) using the pad–dry–cure method, which exhibited only 60.1% of DFP hydrolysis to DHP in 2 h at 32 °C [11].

As reported in the literature, the preparation of detoxification fabrics is difficult as they require the difficult synthesis of catalyst materials, which is complicated and time-consuming, involving processes such as co-electrospinning, CVD, and the sol–gel method [7,8,9,10,11]. Thus, the investigation of a facile fabric detoxification treatment method is worthwhile.

Polyethyleneimine (PEI) is inexpensive and has numerous basic groups. It can act as an organocatalyst for the hydrolysis of nerve agents. Moreover, PEI can be applied to fabrics without inducing any complications compared with other catalysts, such as MOF and guanidine-containing compounds. Microwave curing is known as a more uniform, rapid, and efficient method for fabric treatment compared with conventional heat treatment methods [15,16]. In addition, microwaves are known to form radicals within molecules, thus allowing reactions between molecules without any active reacting functional groups [17,18]. The pad–dry–cure method is a very simple and conventional textile treatment method, and when combined with microwave curing, it allows fast and simple treatment. Therefore, the microwave-assisted pad–dry–cure method can be employed to prepare detoxification fabrics more easily and efficiently than using the co-electrospinning of nylon and a polymer with guanidine groups synthesized from the complicated route, using inorganic catalysts prepared with the sol–gel method followed by thermal annealing, or using the chemical vapor deposition of amine-based chemicals.

**Figure 1:**
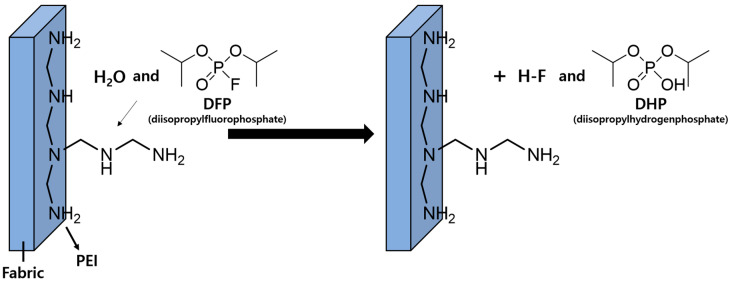
Catalytic hydrolysis of diisopropylfluorophosphate using polyethyleneimine-treated polypropylene fabric as a catalyst.

This study investigates the use of PEI and microwave curing as a facile fabric detoxification treatment to prepare detoxification fabrics against nerve agents. Different concentrations of PEI solutions were prepared and applied to polypropylene using the microwave-assisted pad–dry–cure method. Polypropylene is used in military protective clothing as an air-permeable layer to cover an activated carbon layer to adsorb toxic compounds [19,20]. Thus, the use of detoxification fabrics as a cover is expected to enhance the performance of military protective clothing. The detoxification properties of PEI-treated polypropylene fabrics were evaluated for DFP as a nerve agent simulant (Figure 1).

## 2. Materials and Methods

### 2.1. Materials

Branched PEI (average Mw: ~270,000) and DFP were obtained from Sigma-Aldrich (St. Lousi, MO, USA), whereas ethyl alcohol (99%) was obtained from Daejung Chemical Co., Ltd. (Ansan, Korea). The polypropylene non-woven fabric (spun-bonded, 13 g/m^2^) was provided by the Korea Institute of Industrial Technology.

### 2.2. Preparation of the PEI-Treated Polypropylene Fabrics

The PEI solutions at concentrations of 1 wt %, 5 wt %, and 10 wt % were dissolved in ethyl alcohol. The polypropylene fabrics (10 cm × 10 cm, 0.2 g) were dipped into the PEI solution and then padded using a padding mangle with 100% ± 10% of wet pick-up, so that 0.002, 0.010, and 0.020 g of PEI were treated on polypropylene (10 cm × 10 cm), respectively. Then, the PEI-treated polypropylene fabrics were dried for 30 min at 90 °C and placed in a microwave oven with PEI-treated polypropylene fabrics, which were cured at 1100 W for 2–14 min. The PEI-treated polypropylene fabrics were washed several times with ethyl alcohol and then oven-dried at 70 °C to obtain a constant weight.

### 2.3. Test of the Detoxification Properties of the PEI-Treated Polypropylene Fabrics

The PEI-treated polypropylene fabrics were placed in a 10 mL vial. Then, 0.5 μL of DFP and 25 μL of distilled water were deposited on the prepared samples to allow DFP hydrolysis at 32 °C. The mixture of DFP and DHP was then extracted with distilled water for analysis via gas chromatography (GC). The detoxification properties were evaluated by the decontamination ratio of DFP converted to DHP, which is a nonhazardous compound.

### 2.4. Characterization and Evaluation of the PEI-Treated Polypropylene Fabrics

The chemical structure of the PEI treatment of polypropylene fabrics was characterized using a Fourier-transform infrared (FT-IR) spectrophotometer (Nicolet iS5, Thermo Fisher Scientific., Waltham, MA, USA) equipped with an attenuated total reflection accessory (iD7 ATR, Thermo Fisher Scientific., Waltham, MA, USA). The surface morphologies of both untreated and PEI-treated polypropylene fabrics were observed using a field emission scanning electron microscope (FE-SEM, SU8220, Hitachi, Tokyo, Japan). All samples used for FE-SEM were coated with platinum to improve conductivity before the SEM observation. The X-ray photoelectron spectroscopy (XPS) spectra were obtained using an X-ray photoelectron spectrometer (NEXSA, Thermo Fisher Scientific., Waltham, MA, USA) with a monochromatized Al–Kα source and X-ray spot size of 100 μm. The extracted mixture of DFP and DHP after the hydrolysis reaction of DFP was analyzed via GC (GC-2030, Shimadzu, Kyoto, Japan) with an Elite-1 column (dimethylpolysiloxane, 30 m, 0.25 mm, I.D., 0.25 µm, PerkinElmer) and autosampler (AOC-20, Shimadzu, Kyoto, Japan) to achieve a reliable quantitative analysis.

## 3. Results and Discussions

### 3.1. Optimization of the Microwave Curing Time of the PEI-Treated Polypropylene Fabrics

The PEI-treated polypropylene fabrics were prepared by varying the microwave curing time from 2 to 14 min at 2 min intervals. The DFP decontamination ratios were used to evaluate the optimization of the microwave curing time after performing the DFP hydrolysis reaction on the prepared samples.

**Figure 2:**
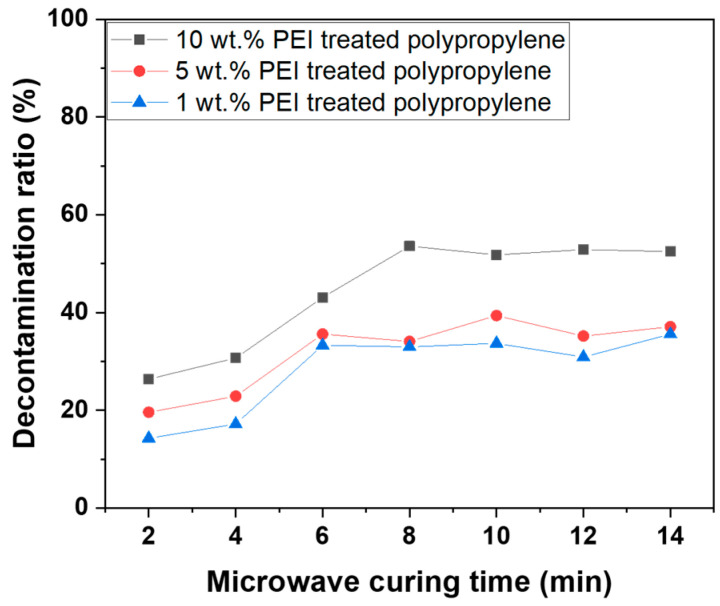
Optimization of the microwave curing time of the polyethyleneimine (PEI) treatment.

Figure 2 presents the results of the microwave curing time optimization. As the microwave curing time increased from 2 to 6 min, the DFP decontamination ratios of the 1 wt %, 5 wt %, and 10 wt % PEI-treated polypropylene fabrics were increased from 14.3% to 33.3%, 19.6% to 35.6%, and 26.4% to 43.1%, respectively. Furthermore, the DFP decontamination ratio of the 10 wt % PEI-treated polypropylene fabric was increased to 53.6% as the microwave curing time increased to 8 min. This result indicates that the amount of PEI that adhered to the polypropylene fabric surface increased when the microwave curing time increased to 8 min, which is an optimum condition for the PEI treatment time.

### 3.2. Chemical and Morphological Changes of the Polypropylene Fabrics

**Figure 3:**
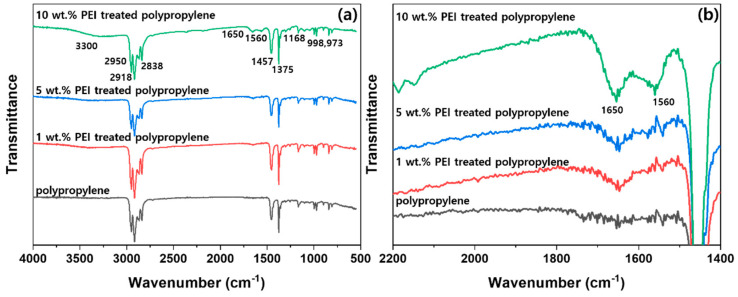
FT-IR spectra of the untreated and PEI-treated polypropylene: (**a**) FT-IR spectrum in the range of 4000–500 cm^−1^; (**b**) expanded FT-IR spectrum in the range of 2200–1400 cm^−1^.

Figure 3 presents the FT-IR spectra of the untreated and 1 wt %, 5 wt %, and 10 wt % PEI-treated polypropylene fabrics. In the FT-IR spectra, the peaks at 2950, 2918, and 2838 cm^−1^ were attributed to the C–H stretching vibration. The peak at 1457 cm^−1^ was attributed to the deformation vibration of CH_2_, whereas the peaks at 998 and 973 cm^−1^ were attributed to the rocking vibration of CH_2_. Furthermore, the peaks at 1375 and 1168 cm^−1^ were caused by the CH_2_ symmetric deformation vibration. These peaks appeared in the polypropylene fabrics [21]. Compared with the untreated polypropylene spectrum, the PEI-treated polypropylene fabric exhibited a new bond due to the −NH stretching vibration, and intramolecular hydrogen bonds appeared at 3300 cm^−1^. However, new peaks attributed to the −NH bending vibration and amide NH bending vibration appeared at 1560 and 1664 cm^−1^, respectively [22], confirming that PEI strongly adhered to the polypropylene fabric surface via microwave curing and remained even after several washing cycles. The peak areas of 1560 and 1664 cm^−1^ in the 1 wt %, 5 wt %, and 10 wt % PEI-treated polypropylene fabrics were 1.74, 3.27, and 10.9, respectively, indicating that the amount of PEI that adhered to the polypropylene fabric surface also increased as the PEI-treated content increased.

**Figure 4:**
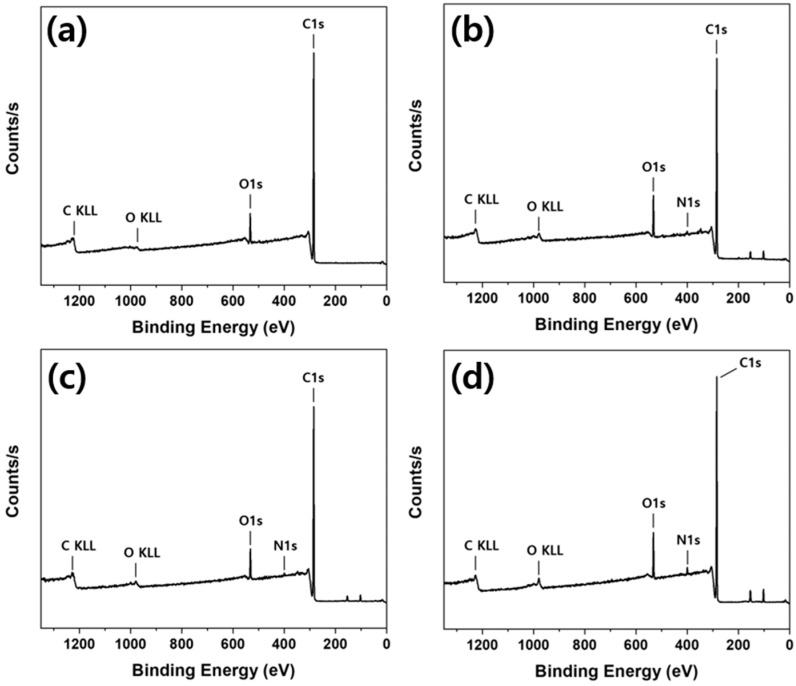
X-ray photoelectron spectroscopy (XPS) survey spectra of the prepared samples: (**a**) untreated; (**b**) 1 wt % PEI-treated; (**c**) 5 wt % PEI-treated; (**d**) 10 wt % PEI-treated polypropylene fabrics.

Figure 4 presents the XPS survey spectra of the prepared samples. The peaks of C1s at 285 eV and O1s at 532 eV can be observed in the untreated polypropylene survey spectrum. The O1s peak appears due to the hydrophilic treatment of polypropylene fabric, as it is composed of only carbon and hydrogen. After the polypropylene fabric treatment with PEI, the new peak occurs at 400 eV, which can be attributed to N1s from PEI.

The surface atomic concentrations based on the XPS survey spectra are presented in Table 1. The surface atomic ratios of N/C ratios of the untreated and 1 wt %, 5 wt %, and 10 wt % PEI-treated polypropylene fabrics were found to be 0, 0.012, 0.019, and 0.024, respectively, indicating that the N/C ratio increased as the PEI-treated content increased.

**Figure 5:**
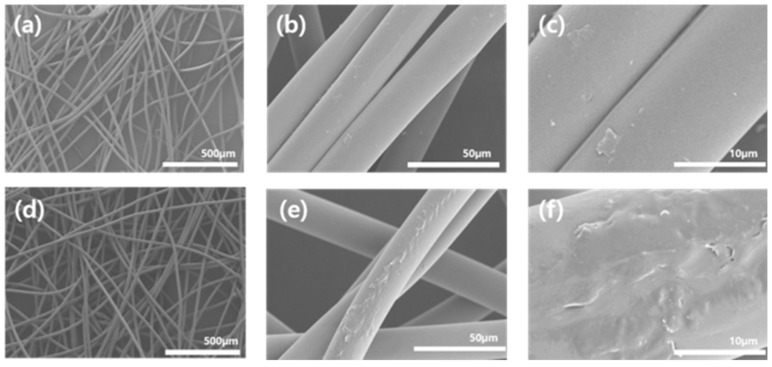
Scanning electron microscope (SEM) images of the untreated and 10 wt % PEI-treated polypropylene fabrics: (**a**–**c**) untreated polypropylene fabric; (**d**–**f**) 10 wt % PEI-treated polypropylene fabric.

Figure 5 presents the SEM images of the untreated and 10 wt % PEI-treated polypropylene fabrics. The surface of the untreated polypropylene fabric was clean and smooth [23]. A thin film was formed on the polypropylene fabric surface after treatment with 10 wt % of PEI on the polypropylene fabric.

**Figure 6:**
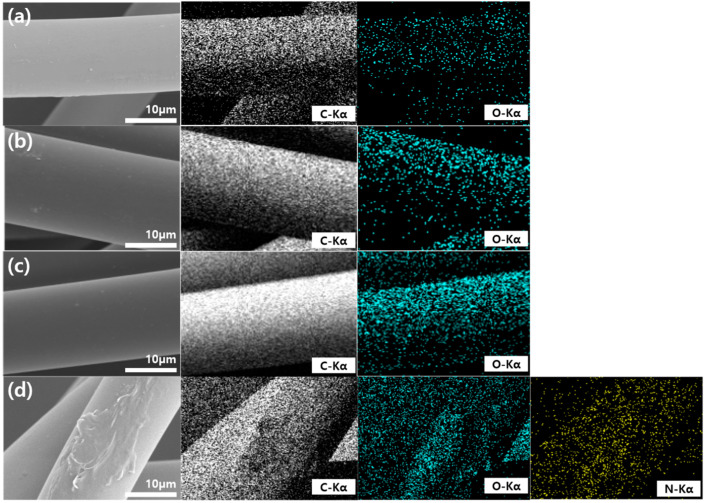
Energy dispersive spectroscopy (EDS) mapping of carbon, oxygen, and nitrogen elements for the untreated and 1 wt %, 5 wt %, and 10 wt % PEI-treated polypropylene fabrics; (**a**) untreated polypropylene; (**b**) 1 wt % PEI-treated polypropylene; (**c**) 5 wt % PEI-treated polypropylene; (**d**) 10 wt % PEI-treated polypropylene.

Figure 6 presents the energy-dispersive spectroscopy (EDS) mapping of the carbon, oxygen, and nitrogen elements for the untreated and 1 wt %, 5 wt %, and 10 wt % PEI-treated polypropylene fabrics. In the untreated polypropylene fabric, the EDS mapping revealed carbon and oxygen distribution. Oxygen appeared due to the oxygen-containing hydrophilic component in the treated polypropylene fabric. The XPS analysis revealed the presence of the nitrogen element in all the prepared samples. However, in the EDS mapping, nitrogen was observed only in the 10 wt % PEI-treated polypropylene fabric. The sample’s surface, with a depth of less than 10 nm, was analyzed via XPS, whereas the bulk surface with a depth of 0.5–5.0 µm was analyzed via EDS. The 1 wt % and 5 wt % PEI-treated polypropylene fabrics exhibited a lower nitrogen content than the carbon and oxygen contents. Thus, the nitrogen contents of the 1 wt % and 5 wt % PEI-treated polypropylene fabrics were not detected in the EDS analysis due to the bulk surface [24].

Table 2 presents an EDS elemental analysis of the untreated and 10 wt % PEI-treated polypropylene fabrics. After the treatment of the 10 wt % PEI solution, the nitrogen content increased from 0 to 6.00, whereas the atomic ratio of N/C increased from 0 to 0.081. This result indicates that PEI sufficiently adhered to the polypropylene fabric surface even after several washing cycles for the 10 wt % PEI-treated polypropylene fabric. Therefore, PEI, which can act as an organocatalyst, can be successfully treated onto the polypropylene fabrics using a facile treatment method combined with the microwave-assisted pad–dry–cure method. Polypropylene does not have an appropriate reaction site to react with PEI, but radicals were formed by microwave curing, and PEI was chemically bound onto the surface of the polypropylene fabric.

### 3.3. Detoxification Properties of the PEI-Treated Polypropylene Fabrics

The detoxification properties of the untreated and PEI-treated polypropylene fabrics were evaluated using DFP, which is a G-type nerve agent simulant. DFP was hydrolyzed to DHP, a nonhazardous compound. However, the boiling point of DHP was higher than the maximum operating temperature of the column’s stationary phase [25]. Moreover, the DHP peak was not detected by the GC analysis. Thus, the decontamination ratio was calculated only based on the DFP peak area. In a previous study, the DFP peak area enabled a reliable quantitative analysis of the decontamination ratio of DFP [26].

**Figure 7:**
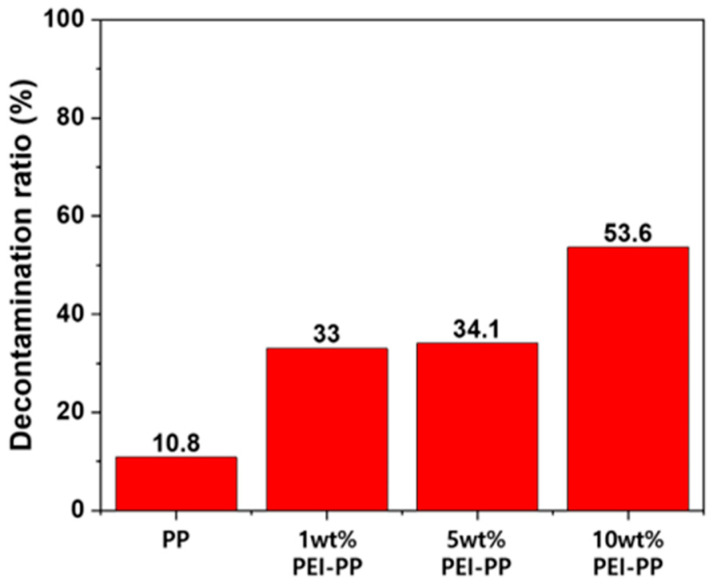
Diisopropylfluorophosphate (DFP) decontamination properties of the prepared samples obtained by gas chromatography (GC).

Figure 7 presents the DFP decontamination ratios of the extracted solution to the DFP hydrolysis for 2 h at 32 °C using the prepared samples. Here, the decontamination ratio of the untreated polypropylene fabric was 10.8%, which was similar to the DFP hydrolysis, without a catalyst for 2 h at 32 °C [13]. The 1 wt %, 5 wt %, and 10 wt % PEI-treated polypropylene fabrics had DFP decontamination ratios of 33.0%, 34.1%, and 53.6%, respectively, indicating that the 10 wt % PEI-treated polypropylene fabric had approximately 1.5 times higher detoxification properties than the other two treatments.

**Figure 8:**
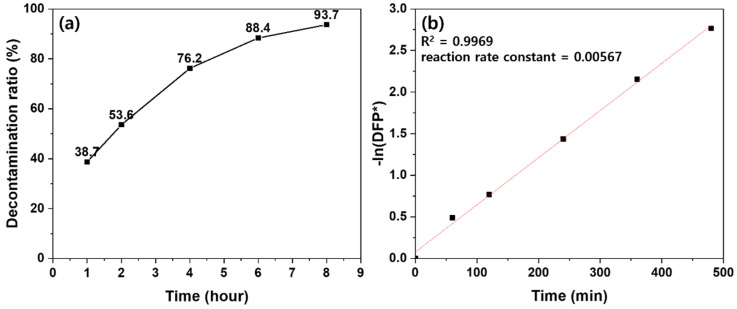
Time profile and kinetic plot for the decontamination of DFP catalyzed by the 10 wt % PEI-treated polypropylene fabric: (**a**) time profile; (**b**) kinetic plot; DFP* is defined as (100 − decontamination ratio of DFP)/100.

**Figure 9:**
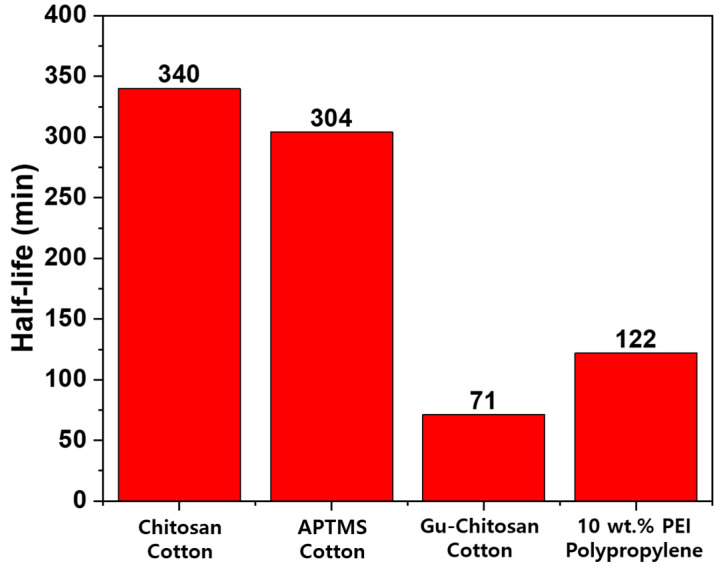
Half-lives of DFP on various detoxification fabrics, which were prepared in this study and in previous studies [13,26]. APTMS: 3-aminopropyltrimethoxysilane.

Figure 8 presents the time profile and kinetic plot for the decontamination of DFP catalyzed by the 10 wt % PEI-treated polypropylene fabric. In Figure 8a, after 8 h, 93.7% of DFP was converted to DHP, which is a nonhazardous compound. Figure 8b demonstrates that the linear correlation coefficient (R^2^) was 0.9969, indicating that it had strong linearity. The half-life was calculated using the reaction rate constant in Figure 8 and compared with the half-lives in the previous studies in Figure 9.

Figure 9 presents the half-lives of DFP on the chitosan, Gu-chitosan, and 3-aminopropyltrimethoxysilane (APTMS)-treated cotton fabric surfaces and the 10 wt % PEI-treated polypropylene fabric surface in the previous studies as well as in this study [13,26]. The half-life of DFP on the 10 wt % PEI-treated polypropylene fabric surface, which was 122 min, was shorter than the half-lives of DFP on the chitosan and APTMS-treated cotton fabric surfaces, which were 340 and 304 min, respectively. The half-life of DFP on the Gu-chitosan-treated cotton fabric surface was 71 min, and the detoxification property of the 10 wt % PEI-treated polypropylene fabric was slightly lower than that of the Gu-chitosan-treated cotton fabric. However, Gu-chitosan was synthesized via the guanidinylation of chitosan, and the detoxification fabric was prepared using an acidic solvent due to the low solubility of Gu-chitosan, which may limit its application to a fabric with low acid stability. Previous studies have demonstrated that the use of catalysts to decrease the half-life of DFP also tends to decrease the half-life of the actual nerve agents. In addition, the mechanism of promoting hydrolysis by the catalyst of the actual nerve agents and that of the simulants was similar [27]. Thus, PEI may be used as a facile fabric treatment agent to prepare detoxification fabrics not only for nerve agent simulants but also for actual nerve agents.

## 4. Conclusions

In this study, the detoxification properties of polyethyleneimine (PEI)-treated polypropylene fabrics were evaluated for their application as detoxification fabrics against diisopropylfluoro-phosphate (DFP), which is a nerve agent simulant. Polypropylene fabric was treated with various PEI concentrations in alcohol solution using a facile fabric treatment process combined with microwave curing. The detoxification properties of the 1 wt % and 5 wt % PEI-treated polypropylene fabrics were found to be 33.0% and 34.1%, respectively. A total of 53.6% of DFP was hydrolyzed to diisopropyl hydrogen phosphate (DHP), a nonhazardous compound, for 2 h at 32 °C by the 10 wt % PEI-treated polypropylene fabric. The half-life of DFP on the 10 wt % PEI-treated polypropylene fabric surface was 122 min, indicating that the PEI-treated polypropylene fabric has comparable detoxification properties for DFP to the other complicated and time-consuming detoxification methods. The basic catalytic effect of PEI exhibited its detoxification properties, and DFP had a short half-life due to the large number of amine groups in PEI. Thus, detoxification fabrics against nerve agents can be prepared more simply, effectively, and quickly by using a facile fabric detoxification treatment method combined with the use of PEI as well as microwave curing than by using complicated methods, such as CVD, organic syntheses from multiple synthetic steps, or inorganic syntheses of catalysts from template methods or sol–gel methods followed by thermal annealing. These fabrics exhibited better detoxification properties than the other detoxification fabrics. Therefore, a facile fabric treatment method has the potential for use to simply and quickly prepare detoxification fabrics against nerve agents.

## Figures and Tables

**Table 1:** Surface atomic concentration of the untreated and 1 wt %, 5 wt %, and 10 wt % PEI-treated polypropylene fabrics based on the XPS survey spectra.

Sample	Atomic Percentage			Atomic Ratios
	C	O	N	N/C
Untreated polypropylene	95.68	4.32	-	-
1 wt % PEI-treated polypropylene	92.85	5.99	1.15	0.012
5 wt % PEI-treated polypropylene	90.66	7.62	1.72	0.019
10 wt % PEI-treated polypropylene	91.34	6.50	2.15	0.024

**Table 2:** A surface atomic concentration of the untreated and 10 wt % PEI-treated polypropylene fabrics based on the EDS analysis.

Sample	Atomic Percentage			Atomic Ratios
	C	O	N	N/C
Untreated polypropylene	95.55	4.45	-	-
10 wt % PEI-treated polypropylene	73.62	20.37	6.00	0.081
